# High sero-prevalence of caseous lymphadenitis identified in slaughterhouse samples as a consequence of deficiencies in sheep farm management in the state of Minas Gerais, Brazil

**DOI:** 10.1186/1746-6148-7-68

**Published:** 2011-11-08

**Authors:** Alessandro S Guimarães, Filipe B Carmo, Marcos B Heinemann, Ricardo WD Portela, Roberto Meyer, Andrey P Lage, Núbia Seyffert, Anderson Miyoshi, Vasco Azevedo, Aurora MG Gouveia

**Affiliations:** 1 Nucleo de Saúde Animal e Microbiologia do Leite, Embrapa Gado de Leite, Rua Eugênio do Nascimento, 610, Juiz de Fora, Postal Code 36038-330, Minas Gerais, Brasil; 2 Departamento de Medicina Veterinária Preventiva, Escola de Veterinária da Universidade Federal de Minas Gerais, Av. Antônio Carlos 6627 Caixa Postal 567, Campus da UFMG, Belo Horizonte, Postal Code 30123-970, Minas Gerais, Brazil; 3 Departamento de Biologia Geral, Instituto de Ciências Biológicas da Universidade Federal de Minas Gerais. Av. Antônio Carlos 6627, Campus da UFMG, Belo Horizonte, Postal Code 30123-970, Minas Gerais, Brazil; 4 Departamento de Bio-Interação, Instituto da Ciência da Saúde da Universidade Federal da Bahia. Avenida Reitor Miguel Calmon, 18 s/n, Vale do Canela, Salvador, Postal Code 40110-100, Bahia, Brazil; 5 Grupo de Extensão da Pesquisa em Ovinos e Caprinos (GEPOC), Belo Horizonte - Brazil

**Keywords:** Caseous lymphadenitis, *Corynebacterium pseudotuberculosis*, sheep, slaughterhouse, Minas Gerais

## Abstract

**Background:**

Caseous lymphadenitis (CLA), caused by *Corynebacterium pseudotuberculosis*, is one of the most important diseases of sheep and goats, causing considerable economic losses for herd owners.

**Results:**

We assessed the seroprevalence of infection with *C. pseudotuberculosis *in 805 sheep from 23 sheep farms that supply slaughterhouses in the state of Minas Gerais; we also analyzed management practices that could be associated with CLA occurrence, used on these and nearby farms that also supplied animals to the slaughterhouse (n = 60). The serum samples for assaying CLA infection were taken at the slaughterhouse. Frequency of infection with *C. pseudotuberculosis *was estimated at 43.7%, and farm frequency was estimated at 100%. Management practices were analyzed through a questionnaire. All farmers (60/60) had extensive/semi-extensive rearing system; 70.0% (42/60) identified sheep individually; 11.7% (7/60) had periodical technical assistance; 41.7% (25/60) disinfected the facilities; 86.7% (52/60) used barbed wire fences and did not implement adequate CLA control measures; only 11.7% (7/60) of breeders reported vaccination against *C. pseudotuberculosis*; 13.3% (8/60) took note of animals with clinical signs of CLA; 1.7% (1/60) opened and sanitized abscesses, and isolated the infected animals; 10.0% (6/60) knew the zoonotic potential of this disease and 1.7% (1/60) of the farmers culled animals in case of recurrence of abscesses.

**Conclusions:**

It can be concluded that *C. pseudotuberculosis *infection is widely spread in sheep flocks in Minas Gerais state in Brazil and that there is a lack of good management measures and vaccination, allowing transmission of this infectious agent throughout the production network.

## Background

Caseous lymphadenitis (CLA) is characterized by abscesses in superficial and visceral lymph nodes. In the superficial form, the peripheral lymph nodes swell and abscess, while in the visceral form there are systemic complications that can lead to chronic thinning [1]. This is a chronic and subclinical disease of sheep and goats distributed worldwide with high prevalence. *Corynebacterium pseudotuberculosis*, its etiological agent, affects sheep and goats, though it can also infect cattle and horses, and rarely, humans. *Corynebacterium pseudotuberculosis *causes human lymphadenitis; most human cases have been classified as occupational infections, affecting workers who have had regular contact with sheep, such as shepherds, shearers, abattoir workers and butchers [2,3]. The bacterium is classified into two biovars, the biovar *ovis*, which mainly affects sheep and goats, causing superficial and visceral abscesses, and the biovar *equi*, which mainly affects horses, causing ulcerating lymphangitis of the distal extremities, ventral abscesses in the thorax and abdomen, and furunculosis [4,5].

In Minas Gerais state, the number of sheep has increased 93.1% in recent years; from 116, 796 in 2000 to 225, 549 in 2008 [6]; this is much greater than in Brazil overall, which recorded a 12.5% increase during the same period [6]. The rapid growth of commercial sheep herds is due to an expansion of sheep meat markets in all regions of the country, though Brazilian production of lamb meat is insufficient to supply consumer demand; consequently, many breeders of beef cattle are now investing in sheep husbandry, with the acquisition of animals from other regions of the country. Commercial sheep production has been increasing in Minas Gerais state of Brazil since 1999, resulting in considerable transit of animals into the state, with acquisition of animals from regions of Brazil where CLA is frequent [6,7]. Sheep husbandry in the Minas Gerais state has not increased more because of a lack of information about production systems and inadequate sheep health regulations. There are insufficient supplies of commercial immunogens for CLA in Brazil, so that systematic vaccination against the etiological agent of CLA has not been possible, even though flock owners consider it the most relevant disease affecting sheep production [8].

CLA was first reported in Brazil in 1972 [9]; however, although it is considered an important sheep disease, with serious economic impacts for farms and industry, only a few epidemiological studies have been carried out in this country. Unfortunately, sheep farmers in the state of Minas Gerais are still unaware of the high prevalence of infection in animals and do not make periodic inspections of animals for CLA. There is little vigilance for clinical disease, resulting in losses in the production of lambs and hides [8-11]. In the only published study on sheep herds, conducted in Minas Gerais state in 2002 [12], animal prevalence was estimated to be 70.9% (95% CI: 64.7-77.0%). The actual prevalence was 75.8%, and the prevalence of farms with infected animals was 95.9% (95% CI: 89.8-98.9%); the ages of the animals ranged from just under four to over 12 months. However, that study did not target the slaughtered animal population. The serological status of the herd is an indication of the presence of the infectious agent and can be used to orient control programs.

We assessed the seroprevalence of infection with *C. pseudotuberculosis *in sheep from sheep farms that supply slaughterhouses in the state of Minas Gerais; we also analyzed management practices used on these farms that could be associated with CLA occurrence.

## Results

**Figure 1:**
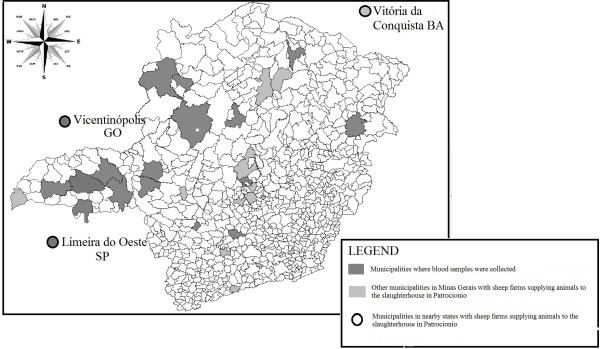
**In Minas Gerais, Brazil, serum samples were collected from sixty municipalities with sheep farms which are suppliers to slaughterhouses including twenty other municipalities with farms therein**. The figure shows all the sixty municipalities with sheep farms which are suppliers to slaughterhouses including twenty other municipalities with farms therein.

The mean number of animals per herd was 35, ranging from 10 to 140. During the study period, 2, 470 sheep were slaughtered and 805 serum samples were collected in the slaughterhouse. Sheep from 23 herds located in 20 different Minas Gerais state municipalities of were thus analyzed (Figure 1).

Serofrequency of *C. pseudotuberculosis *infection among the slaughtered sheep was 43.7% (95% CI: 39.5-47.8%), while the frequency of herds with seropositive sheep was 100% (95% CI: 87.8-100%).

The data obtained using the questionnaires filled out by herd managers are summarized in additional files 1 and 2.

## Discussion

This study was the first made of CLA in sheep herds in Brazil that examined data from serum samples along with management information from slaughterhouse sheep suppliers. The serological results from an earlier study in Minas Gerais [12] and this present study indicate maintenance of a high frequency of seropositive animals and properties; in the first study conducted in 2002, the animal prevalence was 75.8% and prevalence in herds was 95.9%. Our current study, conducted seven years later, showed a high frequency of CLA in herds placed in the same region, demonstrating that CLA is still overlooked by farmers, who have not been taking preventive measures. This lack of disease control constitutes one of the greatest sanitary barriers to the development of sheep husbandry. Among these same animals, 15.3% (data not shown) had caseous nodules in pre-scapular and submandibular lymph nodes, found during slaughter, and 43.7% were seropositive. This shows that most of the infected animals had no clinical signs of CLA. Caseous material from abscesses, the main source of infection, is usually spontaneously drained. Live infectious *C. pseudotuberculosis *has been isolated after five months from areas where abscesses were drained [13].

The estimate of the frequency of animals infected by *C. pseudotuberculosis *in Minas Gerais was within expected limits; the 43.7% prevalence rate in the animals, calculated from the ELISA data is within the estimated confidence interval for frequency, even though 11.7% of sheep owners claimed that they used CLA vaccine. Although sampling and blood collection were conducted properly, there were some problems with the transportation of sera, resulting in the loss of 18 serum samples. All of the sampled herds had at least one infected sheep, showing that CLA is widespread in Minas Gerais, which is the state with the second-largest sheep population in the southeastern region of Brazil [7].

All properties used extensive/semi-extensive management systems (Additional file 1), similar to what was found in 2002 [12]; this is common for farms dedicated to sheep meat production, in which the animals are raised exclusively on pasture during the day, with some protection at night; they graze most of the year. Consequently, the farmers tend to make few inspections of the animals.

In 2002, few farmers made individual identification of sheep (22.7%) [12]. In our current study, only 70% of the farmers made individual identifications, even though it is required by the slaughterhouses. It is possible that the farms that we sampled in this study had a better technological level than the farms sampled in 2002 due to the sanitary requirements that have been adopted by slaughterhouses, such as slaughtering of young animals (less than one year of age), without clinical signs of CLA, in good health and with individual identification. The finding of 30% of flock owners who do not use individual identification of animals appears to be because they think that such control is unnecessary because the sheep will be slaughtered at a young age. However, it is impossible to control livestock or achieve good sanitary control without individual identification.

The high percentage of farmers without any veterinary assistance (Additional file 1) is one of the most serious obstacles impeding successful control of CLA. Only 41.2% of farms sampled in 2002 had veterinary assistance; in our current study the percentage of properties with such assistance was even lower (Additional file 1). Veterinary monitoring is an important determinant for the success of sheep husbandry and it is vital to educate farm workers and establish and maintain sanitary control programs. We can infer that the high seroprevalence of CLA is a consequence of management deficiencies found in 2002 and 2007, among them the low level of veterinary assistance, resulting in serious losses to sheep husbandry and industry.

Few facilities underwent routine disinfection (Additional file 1). Disinfection of facilities is very important because some pathogens, including *C. pseudotuberculosis*, can survive for long periods in the soil. Through experimental contamination of facilities, it was found that *C. pseudotuberculosis *can survive up to eight months at various temperatures [14]. This bacterium has been isolated after five months in places where there has been contamination with pus [14]. Consequently, environmental contamination due to leaking abscess is a real threat, and regular disinfection of facilities is an important measure to control CLA.

The frequent use of fences with barbed wire (Additional file 1), traditional in the state for cattle, is inappropriate for sheep because they tend to force passage between the wires of the fence, thereby suffering lesions in the skin, opening passage for the entry of many bacteria, including *C. pseudotuberculosis*. This is especially important because many of the breeds we sampled animals do not produce wool, including santa ines, morada nova and somalis. Other breeds used locally have only a short light coat of wool and hair, these being dorper and white dorper; there are also close-wool breeds, ile de france and lacaune [6].

Three commercial immunogens for *C. pseudotuberculosis *are available in Brazil; however, only 11.7% of farmers vaccinated their herds systematically against CLA. No farmer sampled in 2002 [12] indicated that he vaccinated sheep against CLA; five years later, the frequency of vaccination remained very low; associated with other poor management practices, this has allowed continued spread of *C. pseudotubercuslosis *in sheep herds in Minas Gerais. Vaccination against CLA is one of the most important measures for disease control.

Very few farmers took notes of animals with clinical signs of CLA, opened, sanitized the abscesses and isolated the animals from the herd, which would be correct management strategy. Most of them let the abscess open by itself or opened it, but did not disinfect and properly drain the wound, and then returned the animal to the herd. Evidently, this procedure does not control, but rather accelerates the rate at which the agent is spread, because the caseous material is a source of contamination of facilities, including troughs, stalls and paddocks. Animals with recurrent abscesses (Additional file 2) should be culled from the herd, but 98.3% (59/60) did not do so. Although animals were individually identified, we found that identification was not used for sanitary control of the herd.

Few farmers were aware that the etiological agent of CLA can affect humans (Additional file 2); this ignorance further increases the risk of human infection. Moreover, only a small percentage of farmers burned and buried the material from abscesses or purulent material from manipulation of clinically-affected animals (Additional file 2), which can also be an important source of infection for other animals and humans. The veterinarian can be key to the orientation of the employees of these properties, teaching them to properly handle the abscesses and discard any material from such manipulations. All of the flock managers reported having information about losses due to CLA during slaughter (Additional file 2); so, they knew the disease is in their herds; but they did not relate this to losses in their farms and risk of human infection.

## Conclusions

These findings support the hypothesis that high seropositivity for *C. pseudotuberculosis *found in sheep in slaughterhouses is related to deficient sanitary management detected in the farms that supplying the animals. The lack of good management measures and of vaccination allows transmission of this infectious agent throughout the production network. Consequently, CLA is widely disseminated in sheep herds in Minas Gerais state and is overlooked by most farmers, favoring endemicity of this disease, resulting in economic losses. Implementation of effective control measures at the property level is important in order to supply lambs with the quality required by domestic and foreign markets.

## Methods

### Sampling

This study was conducted at a sheep slaughterhouse with Federal Inspection Service, in Minas Gerais, Brazil, with visits every 30 days, between July and December 2007; blood samples were collected by jugular vein puncture, randomly; one of each three animals was sampled and the serum was separated and stored at -20°C.

### ELISA

An indirect ELISA using total secreted antigens of *C. pseudotuberculosis *was employed to check for *C. pseudotuberculosis*-specific antibodies in the sera samples collected from the animals received in the slaughterhouse. The specificity and sensitivity were 89% and 99%, respectively [15].

### Questionnaire

A previously validated questionnaire [16], containing questions on animal and herd management and health, was applied to all flock managers by a technician, officer of the industry, responsible for the purchase of sheep for slaughter during the period that the serum samples were collected. Sixty properties, accredited as suppliers of the slaughterhouse, located in 29 municipalities in Minas Gerais were visited. A databank was prepared with data on rearing system, individual identification, technical assistance, disinfection of facilities, use of barbed fence, vaccination against caseous lymphadenitis, records of animals with clinical signs of CLA, management and disposal of materials from abscesses, management of relapsing cases, knowledge of the zoonotic potential of *C. pseudotuberculosis *infection and information concerning loss during slaughter. Animal dentition was not verified because the slaughterhouse only bought animals up to one-year old. Unfortunately, individual herd management information could not be correlated with CLA frequency because slaughterhouse regulations required that the suppliers of such animals not be individually identified, though it was possible to determine that all supplying farms had infected animals.

### Data analysis

Prevalence and confidence intervals were calculated according to [17], with data weighted by the size of flock [17,18]. The real prevalence of infected animals was calculated based on sensitivity and specificity values of 89% and 99%, respectively [13].

## Authors' contributions

ASG, FBC, MBH, APL and AMGG participated in the design of the study, organized the experimental work and wrote the manuscript. RWDP, RM, NS, AM and VA were involved in the design of the study and helped write the manuscript. APL performed the analytical measurements. All authors read and approved the final manuscript.

## Supplementary Material

Additional file 1 **Management practices that could be associated with CLA identified among 60 sheep farms supplying slaughterhouses in the state of Minas Gerais, Brazil, 2007**. The table contains the main management practices associated with caseous lymphadenitis in the sixty sheep farms suppliers to slaughterhouse in Minas Gerais State, Brazil. Click here for file

Additional file 2 **Principal specific CLA preventive measures used on 60 sheep farms supplying slaughterhouses in the state of Minas Gerais, Brazil, 2007**. The table contains the main preventive measures to caseous lymphadenitis in the sixty farms supliers to slaughterhouse in Minas Gerais State, Brazil. Click here for file
