# Supplementary material for: High sero-prevalence of caseous lymphadenitis identified in slaughterhouse samples as a consequence of deficiencies in sheep farm management in the state of Minas Gerais, Brazil
Source: BMC Vet Res. 2011 Nov 8;7:68. doi: 10.1186/1746-6148-7-68 (PMC3256107; doi:10.1186/1746-6148-7-68)
Supplement: Additional file 1 — Management practices that could be associated with CLA identified among 60 sheep farms supplying slaughterhouses in the state of Minas Gerais, Brazil, 2007. The table contains the main management practices associated with caseous lymphadenitis in the sixty sheep farms suppliers to slaughterhouse in Minas Gerais State, Brazil. [file 1746-6148-7-68-S1.DOC]

Table 1 - Management practices that could be associated with CLA identified among 60 sheep farms supplying slaughterhouses in the state of Minas Gerais, Brazil, 2007

| Management practices | n | % |
| --- | --- | --- |
| Extensive/semi-extensive rearing system | 60 | 100.0 |
| Animals individually identified | 42 | 70.0 |
| Technical assistance | 7 | 11.7 |
| Disinfection of facilities | 25 | 41.7 |
| Use of barbed fence | 52 | 86.7 |
