# Supplementary material for: High sero-prevalence of caseous lymphadenitis identified in slaughterhouse samples as a consequence of deficiencies in sheep farm management in the state of Minas Gerais, Brazil
Source: BMC Vet Res. 2011 Nov 8;7:68. doi: 10.1186/1746-6148-7-68 (PMC3256107; doi:10.1186/1746-6148-7-68)
Supplement: Additional file 2 — Principal specific CLA preventive measures used on 60 sheep farms supplying slaughterhouses in the state of Minas Gerais, Brazil, 2007. The table contains the main preventive measures to caseous lymphadenitis in the sixty farms supliers to slaughterhouse in Minas Gerais State, Brazil. [file 1746-6148-7-68-S2.DOC]

Table 2 - Principal specific CLA preventive measures used on 60 sheep farms supplying slaughterhouses in the state of Minas Gerais, Brazil, 2007.

| Management measures | Details | n | % |
| --- | --- | --- | --- |
| Vaccination against CLA |  | 7 | 11.7 |
| Record of animals with clinical signs of CLA |  | 8 | 13.3 |
| Abscess management | Spontaneous drainage | 19 | 31.7 |
|  | Opens, but does not sanitize the abscess before returning the animal to the herd | 34 | 56.7 |
|  | Opens and sanitizes the abscess before returning the animal to the herd. | 6 | 10.0 |
|  | Opens, sanitizes the abscess and isolates the animal. | 1 | 1.7 |
|  | Culling | 0 | 0 |
| Destination of relapsing sheep cases | Culling | 1 | 1.7 |
|  | Treatment | 40 | 6.7 |
|  | No action | 19 | 31.7 |
| Destination of abscesses / purulent material | Bury | 6 | 10.0 |
|  | Burn | 33 | 55.0 |
|  | Burn and bury | 2 | 3.3 |
|  | No action | 19 | 31.7 |
| Aware of zoonotic potential of CLA |  | 6 | 10.0 |
| Information on losses during slaughter |  | 60 | 100 |
| Total |  | 60 | 100 |
